# Engineers’ Moral Responsibility: A Confucian Perspective

**DOI:** 10.1007/s11948-019-00093-4

**Published:** 2019-02-26

**Authors:** Shan Jing, Neelke Doorn

**Affiliations:** 1 School of Humanities, Southeast University, Nanjing, 211189 Jiangsu Province People’s Republic of China; 2 Department of Technology, Policy and Management, Delft University of Technology, P.O. Box 5015, 2600 GA Delft, The Netherlands

**Keywords:** Responsibility, Confucianism, Virtues, Inclusive education, Citicorp Building

## Abstract

Moral responsibility is one of the core concepts in engineering ethics and consequently in most engineering ethics education. Yet, despite a growing awareness that engineers should be trained to become more sensitive to cultural differences, most engineering ethics education is still based on Western approaches. In this article, we discuss the notion of responsibility in Confucianism and explore what a Confucian perspective could add to the existing engineering ethics literature. To do so, we analyse the Citicorp case, a widely discussed case in the existing engineering ethics literature, from a Confucian perspective. Our comparison suggests the following. When compared to virtue ethics based on Aristotle, Confucianism focuses primarily on ethical virtues; there is no explicit reference to intellectual virtues. An important difference between Confucianism and most western approaches is that Confucianism does not define clear boundaries of where a person’s responsibility end. It also suggests that the gap between Western and at least one Eastern approach, namely Confucianism, can be bridged. Although there are differences, the Confucian view and a virtue-based Western view on moral responsibility have much in common, which allows for a promising base for culturally inclusive ethics education for engineers.

## Introduction

Engineering has found its way into all areas of human activity and has become one of the potential driving forces behind global economic development and the creation of new industries. Engineers in various industries contribute to the development of new technologies that influence and shape the way we live, in both anticipated and unanticipated ways (Murphy et al. 2015). Engineers are committed to changing and improving the world. However, engineering may also pose risks for public safety, health and welfare. To protect public safety, health and welfare, engineers are therefore not only responsible for carrying out their work competently and skilfully but they must also be aware of the broader ethical and social implications of engineering and be able to reflect on these issues (Van de Poel and Royakkers 2011; Doorn and Kroesen 2013). It is now widely recognised that this awareness should be cultivated early on in the education of prospective engineers (Zandvoort et al. 2013).

In the 1970s, engineering ethics education first emerged in upper-level engineering education in the US, followed by Germany, the Netherlands, Sweden, France, UK, Canada, Australia, Japan, Russia, China, and other countries (Cao 2015). Rapid changes and the globalisation of the world, including a globalisation of engineering, calls for the internationalisation of education. The American Accreditation Board for Engineering and Technology (ABET) has affirmed the importance of teaching engineering ethics and the 2018–2019 ABET criteria for accrediting engineering programs, for example, define student outcomes in terms of “an ability to design a system, component, or process to meet desired needs within realistic constraints such as economic, environmental, social, political, *ethical*, health and safety, manufacturability, and sustainability” (Criterion 3 sub c; emphasis added) as well as “an understanding of professional and ethical responsibility” (Criterion 3, sub f) (ABET 2017). The process of globalisation compels engineering educators to rethink engineering education in order to educate a better and more rounded type of engineer (Christensen et al. 2015).

Notions of engineering ethics have varied significantly over time, from country to country and across different cultures (Downey et al. 2007, 2015; Newberry et al. 2015). There is a clear need for more instructional resources that place engineering ethics in an international context (Barry and Herkert 2015). Nowadays, many universities and scholars endeavour to make the curriculum more culturally-inclusive (e.g., Crichton and Scarino 2007; Haigh 2009; Ippolito 2007; Jackson 2003). There have been rapid developments within engineering ethical theory (e.g., Davis 1998, 2001; Martin and Schinzinger 2005; Harris 2008; Van de Poel and Royakkers 2011), but the vast majority of these have been set in a Western context (Moriarty 2015). So far, there have only been limited attempts to make engineering ethics education itself more inclusive by paying more attention to non-Western approaches and “inclusive ethics education” has so far received little attention. This paper represents a first attempt to do this.

Given the need for more diversity in engineering ethics education and the globalisation of engineering practice, but also given the context-sensitivity that many engineering ethicists emphasise, it would be interesting to see what a non-Western perspective could add to existing discussions in the engineering ethics literature. This paper focuses on Confucianism as a perspective that has so far not been systematically included in the engineering ethics literature. The current literature on responsibility does not include a systematic study of Confucian moral responsibility. Also, there are hardly any scholarly papers within the engineering ethics literature that discuss Confucian ethics as an approach for discussing and analysing ethical dilemmas in engineering (for exceptions, see Wong 2012; Zhu 2018). This article attempts to redress this imbalance.

It is not suggested that Confucianism can be seen as a proxy for “Eastern philosophy” in general. However, as the former official ideology of the Chinese Empire, Confucianism has had a great influence on the history and culture of many East-Asian countries, including not only China but, due to the presence of a large Han Chinese population, also Taiwan, Korea, Japan, Vietnam and Singapore (Fung 1976). Additionally, Confucian thought focuses on education and the cultivation of virtue, which makes it an interesting perspective to include in engineering education (Riegel 2013).

The aim of the current paper is to explore what a Confucian perspective could add to the engineering ethics literature, specifically focusing on the engineer’s moral responsibility, one of the central concepts of engineering ethics (Doorn and Van de Poel 2012). This will be done by elaborating the topic of responsibility in Confucianism and to examine how it can be used to analyse the well-known case of the ‘Citicorp Building’ from the engineering ethics literature.

By analysing the Citicorp case from a Confucian perspective, this paper aspires not only to contribute to a better understanding of moral responsibility, but also to provide material to be used in the classroom as a basis for studying engineering dilemmas from different cultural perspectives, thereby contributing to more inclusive education.

This research paper includes the following sections: firstly, it provides a brief introduction to the topic of responsibility in the current engineering ethics literature, followed by a section in which we elaborate on Confucian philosophy, including a discussion of the role of responsibility within Confucianism. After that, the Citicorp case is introduced and analysed from the perspective of Confucian philosophy, including a discussion of what a Confucian perspective could potentially add to the existing literature. In the concluding section, we discuss the implications for engineering ethics education.

For the discussion of Confucianism, we draw on the writings of Confucius himself, the disciples of Confucius, and Mencius (a contemporary of Aristotle who lived two centuries after Confucius and who is generally considered to be the most prominent Confucian thinker after Confucius), and the disciples of Mencius.^1^

## Moral Responsibility in Engineering Ethics: From Preventive Responsibility to Responsible Engineering

Responsibility has always been a major theme in engineering ethics literature (Doorn and Van de Poel 2012). For example, the journal *Science and Engineering Ethics* has devoted many papers, including a Special Issue, to the topic of moral responsibility in engineering and technology. In the introduction to this Special Issue, the guest editors linked this relatively strong attention for responsibility in the engineering ethics literature to the context in which engineers work, which does not conform to the assumptions on which the general ethics literature is based. This general ethics literature is often based on possible actions of *individual people* and the *direct consequences* of these actions, where the consequences are assumed to be more or less *certain*. This stands in strong contrast to the actual context in which engineers work. Engineers typically work in collective settings, where, alongside the engineers involved, many different agents jointly shape the technological developments and their social consequences. Secondly, engineering and technology developments are characterised by long causal chains between the actions of engineers and scientists and the subsequent effects that raise ethical concern. Thirdly, the consequences of technology are often characterised by uncertainty and unpredictability (Doorn and Van de Poel 2012, p. 2).

The German philosopher Hans Jonas argued, as early as the late 1970s, for an ethics of responsibility precisely for these three characteristics of engineering and technology development (collectivity, indirect causation and uncertainty) in our technological age, rather than an ethics based on traditional ethical notions such as consequences or duties (Jonas 1984[1979]).

In the literature, a distinction is usually made between backward-looking (or passive) responsibility and forward-looking (or active) responsibility (Van de Poel 2011). Where backward-looking responsibility is relevant in response to an undesirable outcome, forward-looking responsibility is pre-emptive: when nothing has yet gone wrong or if there is the opportunity to behave in a morally praiseworthy way.

This difference between backward-looking and forward-looking responsibility is also reflected in two key discussions within the engineering ethics literature. Until the late 1990s, the focus in engineering ethics was primarily on backward-looking responsibility and questions about alleged wrongdoing: Who can be held responsible for technological failure or disasters? This was often linked to what is generally known as the problem of many hands (Thompson 1980). Backward-looking responsibility requires a number of conditions to be met in order to hold a person responsible.^2^ In many disasters, none of the individuals who are in some way linked to the occurrence of the undesirable event can be said to have fulfilled all these conditions. In this case, no one can be personally held responsible, even though the collective as a whole can be held responsible (Van de Poel et al. 2012). This view on engineering ethics has attracted increasing criticism for being too narrowly focused on wrongdoing and less on the more positive standards that responsible engineering requires (Doorn and Fahlquist 2010; Durbin 2008; Lynch and Kline 2000; Pritchard 2001).

A second strand of literature emerged from this critique of blame-oriented early engineering ethics literature, which started from the forward-looking notion of responsibility and focused on the social or professional responsibility of engineers (Doorn 2014; Harris 2008; Murphy et al. 2015; Van de Poel and Royakkers 2011). In a much-cited paper, the philosopher Charles E. Harris Jr. argued for a break with “preventive ethics,” which has led to a promulgation of negative rules, towards a stronger positioning of engineers’ virtues as “a vehicle for expressing important aspects of engineering professionalism” (Harris 2008, p. 153). In this strand of literature, one of the leading questions became: what attitudes, character traits or virtues should engineers, as professionals, display in order to utilise their professional position or expertise in the realisation of something of value for society?

Some engineering ethicists have tried to develop lists of the virtues or traits that responsible engineers should possess. Inspired by Aristotle’s distinction between moral virtue and intellectual virtue and based on an informal survey among engineers and their managers, the philosopher Pritchard (2001) makes a distinction between the general virtues that all people should possess both within and outside the context of engineering, such as honesty, civic-mindedness, courage, willingness for self-sacrifice, and the virtues that are more closely related to engineering practice, such as cooperativeness (being a good “team player”), the habit of documenting work thoroughly and clearly, commitment to objectivity and commitment to quality. For Pritchard, this second list of virtues contains a necessary but not exhaustive list of conditions for being a morally commendable engineer. One could be a laudable person while only possessing the general virtues, but this would not necessarily result in even basically competent engineering practice (Pritchard 2001, pp. 394–395). Like Pritchard, Harris (2008) divides virtues into technical excellences (non-moral virtues) and non-technical excellences (moral virtues). Technical excellences include those capacities and sensitivities closely related to the technical side of engineering that cannot be expressed well in rules, such as mastery of the relevant aspects of mathematics and physics, engineering science, and design, but also the virtues that allow one to make well-considered decisions in situations of complexity or risk (Harris 2008, pp. 158–159). In addition to these technical excellences, Harris mentions techno-social sensitivity, respect for nature, and commitment to the public good as important non-technical excellences that engineers should exhibit and that should therefore be part of the engineering curriculum.

This brief overview of ongoing discussions in the engineering ethics literature reveals that, although the focus of these discussions has shifted over the past decades, responsibility remains one of the key concepts in the literature. The discussion of responsibility in this new perspective may provide a good starting point should we wish to complement the existing literature with a non-Western perspective.

## Confucian Moral Responsibility

Generally speaking, there has been no clear concept in Confucian ethics which fully corresponds to the notion of moral responsibility (Hansen 1972). However, in Confucian classics, the ideas underlying moral responsibility feature quite prominently. In the last decade, we have seen a rise in papers that study concepts related to moral responsibility from a Confucian perspective, such as papers discussing the concepts of Taoism, harmony, self-cultivation, humanity, family responsibilities, social responsibility, corporate social responsibility, et cetera (Low and Ang 2012, 2013; Wang and Juslin 2009). Huang Yong discusses how Confucianism can deal with the two related issues of virtue ethics and moral responsibility: praise and blame (Huang 2013). The current paper discusses Confucian moral responsibility based on the *Four Books*^3^: the Chinese classic texts illustrating the core value and belief systems in Confucianism.

### The Foundations of Confucian Ethical Theory

Confucianism arose in the patriarchal clan society, and was based on blood ties and established under the framework of Heavenly (the Heavenly Way) and Human (the Human Way) Relations. To understand the foundation of Confucian ethical theory, three characteristics are especially important: blood ties, Heaven, and human nature.

#### Blood Ties

The basis of ancient Chinese civilisation is a strong unity of nature and blood, including totem worship, which reflects the idea that humans are born out of nature and that they naturally worship nature. During the Yellow River civilization in the 3rd and 2nd millennium BC, cities developed as a result of flood control and irrigation of the Yellow River. During this time, political power was also reinforced. This influenced how the unity of nature and blood came to be seen. Nature became less prominent and blood ties became an essential ingredient of Chinese culture, and this ultimately led to the Confucian ethical culture (Chen 2006). Blood ties should be understood in a broad sense. The word that is usually translated in English as ‘country’ is actually composed of two words that are inextricably linked: ‘country’ (guo,国) and ‘family’ (jia, 家). In Chinese culture, the country has always been placed in the position of ‘the big family’. From this etymological perspective, but also from the psychological and emotional perspective of the patriotism of the Chinese nation, it reflects the idea that the ancient Chinese state was developed on the basis of the expanded family clan (Ge 1996). Because of the importance of blood ties, traditional Chinese culture is regarded as a ‘family-country isomorphism’ culture based on filial piety. These blood ties are fundamental to Confucian ethical theory.

#### The Heavenly Way

The word Heaven (tian, 天) has multiple meanings in Confucianism. The first meaning is close to nature as reflected in the following text from *The Analects*: “Does Heaven speak? The four seasons pursue their courses, and all things are continually being produced” (*The Analects*, 19).^4^ The second meaning relates to a higher system of rules, and this is the most important meaning in Confucianism. In this connotation, Heaven is supreme. In *The Doctrine of the Mean,*^5^ nature is presented as ‘what is conferred from Heaven’, where ‘nature’ should be interpreted as human nature. Although Heaven cannot be changed by human power, it can rationally be understood and realised by humans. The ‘Heavenly Way’ can be known through studying human affairs (the lower level), and the study of human life thus becomes the path to understanding the Heavenly Way (the higher level) (Yao 2000).^6^ Furthermore, Mencius pointed out that Heaven inherently possesses goodness, and that the way that humans achieve goodness in the service of Heaven is by “preserving one’s mental constitution” and “nourishing one’s nature” (*Mencius*, 7A).^7^ This evolved from the Heavenly Way into the Human Way.

#### The Human Way

The ‘Human Way’ is based on the goodness of human nature and continuous learning. In Confucianism, an individual should extend him- or herself so as to include others (Fung 1976). In other words, individuals should be as responsible for themselves as they are for others. Why should a person be responsible? Confucius and Mencius attempted to give an answer to this question, and in doing so developed the theory for which they are most famous: that of the original goodness of human nature. According to Confucianism, humans have an inborn nature, which is the basis for goodness. This means that there are good elements in the nature of man. Mencius further developed Confucius’ ideas about human nature in the compilation *Mencius* and attested that human nature is good. He did not claim that all people are born a sage (sheng ren, 圣人), but that there are good elements in human nature. There are also other elements, certainly, neither good nor bad in themselves, that when left uncontrolled can lead to evil. Mencius explained this in terms of what has become known as the four beginnings: “The feeling of compassion is the beginning of benevolence. The feeling of shame and dislike is the beginning of righteousness. The feeling of modesty and yielding is the beginning of propriety. The sense of right and wrong is the beginning of wisdom. Man has these four beginnings (benevolence, righteousness, propriety, and wisdom), just as he has four limbs” (*Mencius*, 6A:6). All people are born with these four beginnings, which, if fully developed, become the ‘four constant virtues’, and lead to responsibility. Mencius gave the example that none of us would fail to be moved if we saw an infant facing imminent death, such as by falling into an open well (Mencius, 2A:6). The reason that we would save the baby is not because we want to have a better relationship with the baby’s parents, nor to get the praise of the neighbours, or because of the despairing cry of the child, but because of our inborn human nature. This became the foundation for Confucian moral responsibility. The moral responsibility of Confucianism does not result from external regulations or coercion, but from the self-consciousness of the individual within human nature. Confucius understood human nature from the ‘genus’: “by nature, men are nearly alike” (*The Analects*, 17:2), by which he means that from birth, all human beings share the same nature, with similar universal characteristics. In the course of their lives, people may become different (“by practice, they get to be wide apart”; *The Analects*, 17:2). Hence, the environment in which people grow up is important, because this environment has a significant influence on how people will develop themselves. Accordingly, different people will undergo self-cultivation in different ways, resulting in a distinction between good and evil.

Within Confucian ethics, continuous learning is important for individuals, which is also reflected in the statement by Tsze-hsia^8^ in *The Analects* that “[a devoted learner is] learning extensively, and having a firm and sincere aim; inquiring with earnestness, and reflecting with self-application; virtue is in such a course” (*The Analects*, 19:6). The benefit is that one can proactively investigate new perspectives, attitudes, and behaviour, and take steps to improve one’s self-cultivation.

### The Classification of Confucian Moral Responsibility

The core structure of Confucianism is ‘saint inside and king appearance’ (nei sheng wai wang, 内圣外王). The text of *The Great Learning*^9^ interrelates moral self-cultivation (saint inside) with general harmony (king appearance) in state and society, and is explained through a chain of achievements and activities. For an individual, once things are investigated (ge wu, 格物), his or her knowledge becomes complete (zhi zhi, 致知). Once a person’s knowledge is complete, his or her thoughts become sincere (cheng yi, 诚意). Once a person’s thoughts are sincere, his or her heart is rectified (zheng xin, 正心). Once a person’s heart is rectified, his or her character becomes cultivated (xiu shen, 修身). Once a person’s character is cultivated, his or her family is regulated. Once the families are regulated (qi jia, 齐家), the State becomes rightly governed (zhiguo, 治国). Once the State is rightly governed, the whole kingdom is made tranquil and happy (tianxiaping, 天下平) (*The Great Learning*). Mencius formulated this chain as follows: “People have this common saying: ‘The kingdom, the State, the family.’ The root of the kingdom is in the state. The root of the State is in the family. The root of the family is in the person of its head” (*Mencius*, 4A:5). This quote reflects an individual’s four levels of moral responsibility: self-responsibility, family responsibility, professional responsibility, responsibility to the universe. Confucian responsibilities are not isolated, but closely related, with progressive characteristics. First of all, Confucian moral responsibility is entirely based on individual self-cultivation, not only for ‘ordinary’ people but for all people: “From the emperor to the monks, you are all based on self-cultivation. The chaos and the final ruler are guilty” (*The Great Learning*). Previously, benevolence (or human-heartedness in other translations of the Chinese texts) was mentioned as the foundation of the goodness of human nature. In Confucianism, this benevolence is linked to the bloodline of human beings, more explicitly to the filial piety of a child to his or her parents.^10^ People should first of all take responsibility for their family, and then extend this responsibility to fulfil their responsibility to others. Thus, responsibility is shown progressively: “He is affectionate to his parents, and lovingly disposed to people generally. He is lovingly disposed to people generally, and kind to creatures” (*Mencius*, 7A:45).

#### Self-Responsibility

Above, the chain of self-responsibility was mentioned, which refers to: investigating things (ge wu, 格物), complete knowledge (zhi zhi, 致知), accomplishing sincerity (cheng yi, 诚意) rectifying one’s heart (zheng xin, 正心), and practicing self-cultivation (xiu shen, 修身). Self-cultivation is the symbol of the saint inside, as it emphasizes an individual’s recognition, training and practice of the ‘four beginnings’. According to Confucianism, taking responsibility is not a result of persecution by external forces, but rather of the individual’s active commitment to his or her own responsibility: “One who restrains himself in order to observe the rites is benevolent. Once you can do this, you will be unanimously considered a man of benevolence. Such a practice wholly depends on the person himself, not on anybody else” (*The Analects*, 12:1). Benevolence (ren, 仁) is the core of Confucian self-cultivation. Self-love is the basic link in the logic of the development of *ren*, insofar as a person with self-love lives a long life of equilibrium (zhong, 中) and harmony (he 和). If a person does not know how to love him- or herself, even if they love someone else, he or she may be hypocritical or have ulterior motives.

Although self-love is a necessary condition, it is not sufficient. People should continue to enhance the realm of benevolence from this starting point and expand it beyond self-love (Han 2016). *Ren* begins with love for one’s own family members, which, embodied as filial piety and fraternal duty, is the essence of *ren*. The basic way to practice *ren* is to apply the principles of loyalty (zhong, 忠) and consideration (shu, 恕) and to treat others as one would wish to be treated by them. The benevolent person cherishes objects and values human life, treats other people as his or her brothers and sisters and other living beings as their fellows. The benevolent person respects and serves the spirit and makes other people incline towards honesty, kindness and other important virtues,^11^ manifesting the essence of humanity. In other words, to practice self-cultivation with benevolence means taking on responsibilities in the family, in one’s profession and in the universe.

#### Family Responsibility

Confucianism views the family as the natural habitat of humans, and treats the family as the key unit in human society (Moise 1986). Family, as the most basic and intimate relationship, occupies an important and fundamental position in Confucianism, where the notion of family—unlike most western interpretations—does not contain clear boundaries between those who belong and those who do not belong to the family. Fei Xiaotong, a well-known Chinese sociologist, described traditional Chinese human relationships, including family, as: “[…] circles that appear on the surface of a lake when a rock is thrown into it. Everyone stands at the centre of the circles produced by his or her own social influence” (Fei 2012). Hence, family can be closer or further away, but it is more a matter of sliding scales than a clearly demarcated category.

Family responsibility refers to the concept of family-regulating (qi jia, 齐家) as in the text of *The Great Learning*. To achieve unison within one’s own family, it is of great importance to establish the moral responsibility of the family members. *The Book of Rites*^12^ mentions that “What are the things which men consider right? Kindness on the part of the father, and filial duty on that of the son; gentleness on the part of the elder brother, and obedience on that of the younger; righteousness on the part of the husband, and submission on that of the wife; kindness on the part of elders, and deference on that of juniors; with benevolence on the part of the ruler, and loyalty on that of the minister—these ten are the things which men consider to be right” (*The Book of Rites*, 7(2):19). That is to say, family members, especially the core members of the family, should handle the triple relationship: the parent and child relationship, the partner relationship and the relationship between young and old. ‘Filial Piety’ (xiao, 孝) and ‘Brotherhood’ (ti, 悌) are the two most important values of the familial relationship. In the relationship between parents and children, Confucianism emphasises the filial piety of children with respect to their parents. This piety of children towards their parents is enabled by the support of their parents. “Kindness on the part of elders, and deference on that of juniors” (*The Book of Rites*, 7(2):19) is not just the ethical requirement to deal with the relationship between people in family relations, but also the ethical requirement to deal with the relationship of peers outside the family. Specifically, elders should be friendly to young people and the young should respect the elderly people. These ethical requirements for handling family relationships are also the respective responsibilities of family members. As in the discussion of self-responsibility, the effect of family responsibility transcends family relationships, as reflected in this quote by Mencius: “If each man would love his parents and show the due respect to his elders, the whole land would enjoy tranquillity” (*Mencius*, 4A:11).

#### Professional Responsibility

Beyond the family, people also have a professional responsibility, which is clearly illustrated in the following quote by Confucius: “The mechanic, who wishes to do his work well, must first sharpen his tools” (*The Analects*, 15:10), where the word ‘tool’ is intended as a metaphor for both work (technical) skills but also to self-responsibility. The influential contemporary Confucian thinker Fung Yu-lan (冯友兰), discussed professional moral responsibilities in his book *A New Treatise on the Nature of Man* (*Xinyuanren, 新原人*). Fung Yu-lan argues that individual people cannot live without society. All people have a place in society, where they should “assume professional responsibility to the greatest extent so that human beings can live in harmony (jin xing zhi ming, 尽性至命)” (Fung 2007). Professional responsibility is achieved through virtues such as reverence, loyalty, courage, and honesty.^13^ By fulfilling their own self-responsibility, people also work for the benefit of society at large. According to Confucianism, no matter what kind of work a person is engaged in, one should work in one’s place, in one’s government, in one’s devotion to duty, and in diligent work. When in a position of responsibility, one should not ease off, but approach one’s work with loyalty.^14^ A clear example of how far this professional responsibility may go can be found in Mencius’ example of Da Yu (Mencius, 3A:4). The Emperor appointed Yu to manage the floods in the Yellow River. Yu was so dedicated that he was said to have passed his home three times without entering. Ultimately, it took Yu and his colleagues 13 years of continuous effort before they succeeded in dredging all the rivers, and in doing so, were able to manage the floods effectively. Mencius explained the dedication of Yu in terms of what would have happened if he had not assumed responsibility; many people in the kingdom would probably have been drowned during a flood. Hence, professional responsibility, even though it stems from self-responsibility and family responsibility, may come with great personal gains and losses.

#### Responsibility to the Universe

Lastly, in Confucianism, people have a responsibility to the universe, which includes all living creatures. Mencius recognised that people have a responsibility to animals as well, even if they were labelled ‘inferior’: “In regard to inferior creatures, the superior man is kind to them, he is affectionate to his parents, and lovingly disposed to people generally and kind to creatures” (*Mencius*, 7A:45) and “So is the superior man affected towards animals, having seen them alive, he cannot bear to see them die; having heard their dying cries, he cannot bear to eat their flesh; therefore, he keeps away from his slaughter-house and cook-room” (*Mencius*, 1A:7). In *The Doctrine of the Mean*, responsibility to the universe is explained in terms of the natural unity of all living things. In this regard, people have the responsibility not to interfere with, or to disrupt or destroy natural ecology, which is generally known as ‘The Harmonious Relationship between Man and Nature’ (min bao wu yu, 民胞物与), a term coined by Chang Tsai^15^ during the North Song Dynasty.

### Reception of Confucianism in Modern China and Current Debates

Although Confucianism is still one of the most influential Asian world views, two points in particular have prompted debate in the current literature.

#### The Place of Confucianism in Contemporary Society

Confucianism’s ‘saint inside and king appearance’ was in line with the feudal system of ‘family-country isomorphism’ culture based on filial piety (Xu 2017). Therefore, in Chinese history, Confucianism was not just a simple philosophy or religion, but a set of ideologies that comprehensively arranged human civilisation. Not only the individual’s daily life, but also the family and the country were all within the scope of Confucianism (Yu 1998). In other words, Confucianism not only regulated and guided the moral life of the people, but was also embedded in various political, economic, and educational systems in order to realise its value. With the collapse of the Chinese feudal system, some scholars fear that Confucianism, now having has lost its support system, has become a ‘wandering soul’ (Yu 1998). In view of the predicament of Confucianism, the Chinese American historian and Sinologist Yu Yingshi argues for a stronger integration of contemporary Confucianism into daily life in order to realise the value of Confucianism. The perception of a mismatch between Confucian ethics and modern Chinese society is a topic of continued disagreement. Some argue that, despite its broken relationship with China’s modern political system, Confucianism is still reflected in China’s social systems, including the family and education systems (Xu 2017). For example, China’s law on the Protection of the Rights and Interests of the Elderly regulates the responsibility of “Maintenance and Support by the Family”. Since 2013, family members living separately from the elderly are obliged to visit the old folk frequently, and risk a legal penalty if they fail to do so. As a practical theory, Confucianism is strongly entrenched in the education system, with a strong focus on moral cognition and behaviour training (Xu 2017; Zheng 2016), which may also find its way into engineering ethics education.

#### *The Paradox of* Filial Piety (*xiao*, *孝*) *and* Benevolence (*ren*, *仁*)

A famous story in *The Analects* discusses the tension between filial piety and benevolence. In this story, the Duke of Sheh mentions the case of a father who has stolen a sheep. The son, who knows of the theft, reports the theft, thereby transgressing his duty of filial piety. Confucius replies by saying that the right action here would be for a father to conceal his misconduct from his son and for the son to conceal the misconduct of his father.^16^ This story has been extensively studied by the academic community and is known as “Mutual Concealment among Family Members” (Guo and Zhang 2015). This story has encouraged some scholars to believe that Confucianism has fallen into a deep paradox: on the one hand, Confucius regards filial piety as the basis of benevolence, and requires people to proceed from the starting point of blood relatives in order to promote benevolent love; on the other hand, to ensure that filial piety remains fundamental in difficult times, one has to give filial piety a sacred meaning, and it even asks people to deny benevolence in order to maintain filial piety (Deng 2010; Liang 2012; Liao 2013; Liu 2005, 2012). Some scholars have argued that it is reasonable in this context to choose mutual concealment, as it does not imply that one justifies the fault itself or that one should refrain from investigating the truth and making judgments. By concealing, one can maintain a good family relationship, which in turn provides a solid foundation for benevolence and harmony (Guo 2004, 2011).

Interestingly, although this point of Confucianism has received criticism in the ethical literature, ‘mutual concealment’ is legally recognised in many jurisdictions through spousal testimonial privilege.

### Comparison of Confucianism with Other Ethical Theories

A comparison of the foundations of Confucian ethical theory and Confucian moral responsibility with other philosophical traditions suggests some commonalities but also differences. The Confucian idea of innate willingness to do the good thing resembles Kantian good will. Likewise, for Kant, a good will is good under all conditions; that is, it should be good in itself and not be good by virtue of how it affects an agent’s own happiness or by virtue of any other effects it may or may not produce. A good will would still “shine like a jewel” even if it were “completely powerless to carry out its aims” (Kant 2011[1785], p. 394). Also, the focus on reasoning as a way to understand the Heavenly Way shows clear similarities with Kantian thought. However, contrary to Kantian good will, the Confucian willingness to do good is linked to virtues which stem from the four beginnings. Also, the Confucian focus on ‘filial piety’ stands in contrast to Kant’s universalist ethics (for which Confucianism has also received criticism, as discussed above).

The attention paid to virtues and the focus on self-cultivation in Confucian thought show strong similarities with virtue ethics. But here again, Confucian virtues cannot simply be seen as carbon copies of Aristotelian virtue ethics. The distinction between moral and intellectual virtues would be artificial within Confucianism. Most Confucian virtues ultimately amount to carefulness and sincerity in what one does, aimed at harmony and keeping a balance. Clear similarities between Aristotelian virtue ethics and Confucian thought can be found in the way one can become a virtuous person. Where Aristotelian virtue ethics focuses on practical wisdom and becoming virtuous by following good examples, Confucianism maintains the study of human life and human affairs as a path to understanding the Heavenly Way.

In order to gain insight in how a Confucian perspective might enrich the engineering ethics literature, the next section will discuss a specific case that is often used in the engineering ethics literature and analyse it from a Confucian perspective.

## Case: Citicorp Building

### Motivation of Case

In this section, we discuss the case of the Citicorp Building. It is a well-known case in the engineering ethics literature. Interestingly, the case is discussed both in the literature as an instance of the problem of many hands (Van de Poel and Royakkers 2011) and as an illustration of how a virtuous engineer responds when he or she discovers insufficiencies in the construction work (Harris et al. 2008; Martin and Schinzinger 2005; Pritchard 2001; Whitbeck 1998). This praise is not unanimous: although most of the literature assesses the behaviour of LeMessurier as praiseworthy, it has also been described as one of concealment where the main actor did not do all he could to inform the public about the potentially dangerous situation (Kremer 2002).

### Case Description^17^

Structural engineer Bill LeMessurier designed the Citicorp Center in Manhattan, built in 1977. He faced the challenge that St. Peter’s Lutheran Church owned and occupied a corner of the lot that had been designated in its entirety as the site for the new structure. He created an innovative design, with the building set on four pillars, nine floors high, placed at the centre of each side of the building, and the church would be offered a brand-new St. Peter’s, standing freely underneath one of the cantilevered corners.

In 1978, some questions posed by an engineering student prompted LeMessurier to review certain structural aspects of the tower. He wondered if the structure could withstand certain loads caused by strong quartering winds. He called his colleague who was in charge of the tower erection, only to discover that the joints had been bolted together rather than welded, on the advice of supplier Bethlehem Steel, due to the high costs of welding. LeMessurier came to the conclusion that a 16-year storm (one that passes every 16 years) could conceivably rip one of the connections loose causing the entire building to collapse. A Red Cross estimate indicated that if the building collapsed, up to 200,000 people could lose their lives.

LeMessurier had a solution to this problem. However, he faced an ethical dilemma involving a conflict between his responsibilities to ensure the safety of his building and the people who used it, his responsibilities towards various financial constituencies, and his self-interest, which might be served by remaining silent. Finally, he decided to inform the lawyers, insurance companies, the chief architect, the chief executive at Citicorp Center and the city hall. Contrary to his expectations, all parties were highly cooperative and the corrections that LeMessurier advised were carried out. The building is now much safer than it was when the original plans were followed during its original construction.

In many texts on the responsibility of engineers, LeMessurier is praised for demonstrating how courage, honesty and concern for safety are implemented in engineering practice.

### Discussion

Pritchard uses the case Citicorp Building and LeMessurier’s conduct in particular to illustrate the list of important virtues an engineer should display (Pritchard 2001). Firstly, he had the courage to report the error, even though it could have considerably damaged his reputation to do so. Secondly, he had both integrity and honesty. When he came to the conclusion that a 16-year storm could potentially rip one of the connections loose and the entire building could collapse, he did not conceal the fact, but chose to admit the problem and to actively find a solution. He also demonstrated another virtue: willingness to make a self-sacrifice for the safety of the public. We know that if the building had collapsed, up to 200,000 people could have lost their lives. LeMessurier, in reporting the error, was willing to assume the risk of damaging his reputation and having to compensate for the loss. In addition to these moral virtues, Pritchard also recognizes intellectual virtues in LeMessurier’s behaviour, particularly an ‘openness to correction’ and a ‘commitment to quality’.

However, in a critical essay presented at the “Ethics and Architecture” conference on 6 April 2002 in New York, the architect Eugene Kremer presents a different analysis of the case. For Kremer, LeMessurier’s behaviour was not as laudable as most of the engineering ethics literature presents it. Kremer is especially critical about LeMessurier’s lack of openness about the case. It took almost two decades before LeMessurier presented his experience to his peers in the engineering community, which Kremer sees as a transgression of the profession’s commitment to advance the knowledge and usefulness of the profession (Kremer 2002).

What form would an analysis from a Confucian perspective take? In the ‘saint inside, king appearance’ structure of Confucian ethics, self-cultivation has been treated as the symbol of saint inside. LeMessurier exhibits good self-cultivation; taking responsibility was not a result of pressure by external forces, but rather of the active commitment to his own responsibility as an engineer. From this perspective, LeMessurier can be considered to be a man of benevolence, committed to the safety of 200,000 people.^18^

### Confucian Addition to the Engineering Ethics Literature

So far, the analysis is broadly similar to those found in most of the engineering ethics literature. Where a Confucian approach might provide a new perspective is in the evaluation of reputation and shame.

Focusing specifically on professional responsibility, we already remarked that the Aristotelian distinction between moral virtues and intellectual virtues is not explicitly made in Confucianism. Professional responsibility in Confucianism is therefore seamlessly linked to a person’s other responsibilities. Despite the lack of explicitly labelled intellectual virtues, one could still recognise some virtues that are specifically linked to professional responsibility and that resemble Aristotelian virtues, notably reverence, loyalty, courage, and honesty.^19^ Ultimately, these virtues should be instrumental in attaining harmony (jin xing zhi ming, 尽性至命) (Fung 2007), which is also reflected in the notion of righteousness that is associated with feelings of shame and dislike. Where Kremer is critical about LeMessurier’s behaviour because he remained silent after the flaw in the construction was repaired, the Confucian focus on harmony and avoidance of shame may praise LeMessurier’s behaviour after the repair exactly *because* he was able to avoid damaging his reputation, not only for himself but also for the company.

This does not mean that Confucianism does not know self-sacrifice. In his analysis of the Citicorp case, Prichard points out that one should be willing to make self-sacrifice (Pritchard 2001), but Confucius also said that “the determined scholar and the man of virtue will not seek to live at the expense of injuring their virtue. They will even sacrifice their lives to preserve their virtue complete (sha shen yi cheng ren, 杀身以成仁)” (*The Analects*, 15:9). However, self-sacrifice should be instrumental in achieving harmony and not as a goal in itself.

In the existing discussions about this case in the engineering ethics literature, relatively little has been said about the responsibility of anyone apart from LeMessurier or the responsibilities of LeMessurier towards others, including Citicorp itself (Pritchard 2001). Here the notion of Confucian family responsibility may especially be helpful. As explained above, Confucianism views the family as the natural habitat of humans. In China, a family includes not only the husband, the wife, and the minor children, but also a wider group of relatives that can be expanded to include even more people. At the same time, family responsibility is a reminder that our primary responsibilities are to those closest to us, and only then can we seek solutions that extend our responsibilities outward to others. So in our case, from the perspective of Confucianism, LeMessurier not only took responsibility for his own family, but also protected the greater ‘family’ associated with Citicorp. But other people also have responsibilities. Everyone shares a responsibility to keep societal order intact. The term commonly used here is *Ren lun* (人伦), which can be translated as human relationships. The term *lun* (伦) consists of two components, ‘human’ (ren, 亻) and ‘order’ (lun, 仑).

A very preliminary observation that may be worth studying empirically is whether this structure of human relationships in Confucianism is more conducive to taking active responsibility, as all people bear some responsibility, or whether it may actually be more susceptible to the problem of many hands, exactly because of the shared responsibilities. After all, if everyone has a certain degree of responsibility, no one may actually feel responsible for the problems (cf. the bystander effect). Here it may be interesting to investigate whether there are clear instances of the ‘problem of many hands’ in cultures which are influenced by, for example, Confucianism.

## Concluding Remarks

In discussing the similarities and differences between Confucian moral responsibility and the notion of responsibility in the existing engineering ethics literature, the comparison suggests the following. On the most abstract level, responsibility in Confucianism is aimed at harmony and all actions are ultimately instrumental to achieving harmony. When compared to virtue ethics based on Aristotle, Confucianism focuses primarily on ethical virtues; there is no explicit reference to intellectual virtues. It was also shown that virtues should be seen in their original context, as they may have had a quite different meaning. For example, courage in Confucianism is very different to courage in ancient Greek culture.^20^

An important difference between Confucianism and most Western approaches, which becomes especially apparent in the concept of family, is that Confucianism does not have clear boundaries marking where a person’s responsibility ends. Moral responsibility extends from close relations to people further away, but it has no defined limits. This may be conducive to a culture of active responsibility, but it may at the same time introduce opaqueness in the distribution of responsibilities.

So far the comparison between Confucianism and Western approaches to responsibility; what could this Confucian perspective add to the engineering ethics literature, or more specifically, how should inclusive engineering ethics education be developed? This paper started with the observation that engineering ethics education is currently rather narrowly focused on Western approaches. Our analysis suggests that the gap between Western and at least one Eastern approach, namely Confucianism, can be bridged. Although there are differences, the Confucian view and especially a virtue-based Western view on moral responsibility have much in common, which allows for a promising base for culturally inclusive ethics education for engineers.

However, if the commonalities are emphasised too strongly, there is a risk of using similar terms for concepts that have quite different meanings. The differences in interpretation of the concept of family was already mentioned, but the same applies for the listed virtues, which may have quite different meanings in different cultures. If Confucian texts are used in the classroom, it may be good to be aware of this to reduce the risk of mainstream Western approaches colonising non-Western concepts.

## Footnotes

1 This paper tries to use as much as possible gender-neutral language. However, in order to stay true to the original texts, we use the gender pronoun as used by Confucius himself or his followers when referring to these texts.

2 Although academics disagree on the precise formulation, the following conditions capture the general notion of when it is fair to hold an agent morally responsible for (the consequences of) their actions: moral agency, voluntariness or freedom of action, knowledge of the consequences of one’s actions, causality, and transgression of a norm (Doorn 2012; see also Bovens 1998; Feinberg 1970; Fischer and Ravizza 1993).

3 *Four books* are four pre-Qin Chinese classic texts, including *The Analects* (*Lunyu*, 论语)*, The Great Learning* (*Daxue*, 大学)*, The Doctrine of the Mean* (*Zhongyong*, 中庸) and *Mencius* (*Mengzi*, 孟子). They were selected by Zhu Xi during the Song Dynasty to serve as a general introduction to Confucian thought. Use is made of the annotations of the *Analects* (Yang 2006) and of *Mencius* (Yang 2008). For the English translations, use has been made of Legge (1861–1872).

4 *The Analects* (*Lunyu*, 论语) is the compilation of speeches by Confucius and his disciples.

5 *The Doctrine of the Mean* (*Zhongyong,* 中庸) was originally a chapter in *The Classic of Rites*. The authorship of *The Doctrine of the Mean* is controversial. It is said to be written by Tzu Ssu (Zi Si, 子思) a disciple of Zeng Shen, but it is also maintained that it was written by Confucians during the Han Dynasties.

6 Cf. Mencius’ quote that “He who has exhausted all his mental constitution knows his nature. Knowing his nature, he knows Heaven” (*Mencius* 25.1).

7 *Mencius* (*Mengzi*, 孟子) was probably compiled by Mencius, his disciples or disciples of his disciples. The received text of the Mengzi is divided into seven ‘books,’ each of which is subdivided into two parts (labeled ‘A’ and ‘B’ in English), and then further subdivided into ‘chapters.’ As a result, a passage can be uniquely identified in any translation; for example, 1A1 is the first passage in any edition or translation of the text and 7B38 is the last. See also the entry on Mencius in the Stanford Encyclopedia of Philosophy (https://plato.stanford.edu/entries/mencius/).

8 Tsze-hsia (Zixia, 子夏), one of the most important disciples of Confucius, makes most of the pronouncements in section 19 of *The Analects*, broadly covering many of Confucius’ main ideas of humaneness, the gentleman, and fondness for learning.

9 *The Great Learning*, originally a chapter in *The Classic of Rites,* was probably written by Zeng Shen, a disciple of Confucius.

10 Cf. “the filial piety is also the foundation of benevolence based on the bloodline of human beings” (*The Analects*, 1).

11 The main Confucian virtues are: benevolence (仁, ren), righteousness (义, yi), courtesy or politeness (礼, li), wisdom (智, Zhi), trustworthiness (信, xin), courage (勇,yong), filial piety (孝, xiao), brotherhood (悌, ti), loyalty (忠, zhong), consideration (恕, shu), reverence(敬, jing), honesty (诚, cheng), character And Integrity (廉, lian), shamefulness (耻, chi).

12 *The Book of Rites (Liji*, 礼记*)* is a core text of the Confucian canon edited by Dai Sheng. The work consists of 49 chapters, outlining the social forms, administration and ceremonial rites of the Zhou Dynasty. Dai Sheng (dates unknown), also known as Xiao Dai, was a Confucian scholar and expert of the ritual classics who lived in the early Former Han Dynasty. Use has been made of the English (Legge 1861–1872).

13 Cf. “Let the superior man never fail reverentially to order his own conduct” (*The Analects*, 12:5); “It is, in retirement, to be sedately grave; in the management of business, to be reverently attentive; in intercourse with others, to be strictly sincere” (*The Analects*, 13:19) and “In regard to his doing of business, he is anxious that it should be reverently careful” (*The Analects*, 16:10).

14 Cf. Confucius’ response to his disciple Tsze-chang who asked how to engage in politics: “The art of governing is to keep its affairs before the mind without weariness, and to practise them with undeviating consistency” (*The Analects*, 12:14).

15 Chang Tsai (Zhang Zai, 张载) (1020–1077), a realist philosopher of the Song Dynasty, was a pioneer in giving neo-Confucianism a metaphysical and epistemological foundation.

16 Cf. “Among us, in our part of the country, those who are upright are different from this. The father conceals the misconduct of the son, and the son conceals the misconduct of the father. Uprightness is to be found in this” (*The Analects*, 13:18).

17 The description of the case is based on Martin and Schinzinger (2005), Morgenstern (1995), and Pritchard (2001).

18 Cf. the passage from The Analects mentioned in “The Heavenly Way” Section, where Confucius described a practice of benevolence as one which “wholly depends on the person himself, not on anybody else” (*The Analects*, 12:1).

19 For example, Confucius stated that, “In regard to his doing of business, [one should be] anxious that it should be reverently careful” (*The Analects*, 16:10).

20 For example, in Ancient Greek, courage is generally regarded as an independent virtue. However, in Confucianism, courage must defer to other virtues: “… without propriety, courage of a man will lead to insubordination” (*The Analects*, 8:2) and “those who love courage and hate poverty would be insubordinate” *(The Analects*, 8:10) (Jiang 2012).
